# Glomerular filtration rate in critically ill neonates and children: creatinine-based estimations versus iohexol-based measurements

**DOI:** 10.1007/s00467-022-05651-w

**Published:** 2022-08-02

**Authors:** Nori J. L. Smeets, Esther M. M. Teunissen, Kim van der Velden, Maurice J. P. van der Burgh, Demi E. Linders, Elodie Teesselink, Dirk-Jan A. R. Moes, Camilla Tøndel, Rob ter Heine, Arno van Heijst, Michiel F. Schreuder, Saskia N. de Wildt

**Affiliations:** 1 Department of Pharmacology and Toxicology, Radboud Institute for Health Sciences, Radboud University Medical Center, PO Box 9101, 6500 HB Nijmegen, Netherlands; 2 Intensive Care and Department of Pediatric Surgery, Erasmus MC Sophia Children’s Hospital, Rotterdam, Netherlands; 3 Department of Clinical Pharmacy & Toxicology, Leiden University Medical Centre, Leiden, Netherlands; 4 Department of Pediatrics, Haukeland University Hospital, Bergen, Norway; 5 Department of Pharmacy, Radboud Institute for Health Sciences, Radboud University Medical Center, Nijmegen, Netherlands; 6 Division of Neonatology, Department of Pediatrics, Amalia Children’s Hospital, Radboud University Medical Center, Nijmegen, Netherlands; 7 Division of Pediatric Nephrology, Department of Pediatrics, Amalia Children’s Hospital, Radboud University Medical Center, Nijmegen, Netherlands

**Keywords:** Acute kidney injury, Augmented renal clearance, Iohexol, Glomerular filtration rate, Creatinine, Creatinine clearance

## Abstract

**Background:**

Acute kidney injury (AKI) and augmented renal clearance (ARC), both alterations of the glomerular filtration rate (GFR), are prevalent in critically ill children and neonates. AKI and ARC prevalence estimates are based on estimation of GFR (eGFR) using serum creatinine (SCr), which is known to be inaccurate. We aimed to test our hypothesis that AKI prevalence will be higher and ARC prevalence will be lower in critically ill children when using iohexol-based measured GFR (mGFR), rather than using eGFR. Additionally, we aimed to investigate the performance of different SCr-based eGFR methods.

**Methods:**

In this single-center prospective study, critically ill term-born neonates and children were included. mGFR was calculated using a plasma disappearance curve after parenteral administration of iohexol. AKI diagnosis was based on the KDIGO criteria, SCr-based eGFR, and creatinine clearance (CrCL). Differences between eGFR and mGFR were determined using Wilcoxon signed-rank tests and by calculating bias and accuracy (percentage of eGFR values within 30% of mGFR values).

**Results:**

One hundred five children, including 43 neonates, were included. AKI prevalence was higher based on mGFR (48%), than with KDIGO or eGFR (11–40%). ARC prevalence was lower with mGFR (24%) compared to eGFR (38–51%). eGFR equations significantly overestimated mGFR (60–71 versus 41 ml/min/1.73 m^2^, *p* < 0.001–0.002). Accuracy was highest with eGFR equations based on age- and sex-dependent equations (up to 59%).

**Conclusion:**

Iohexol-based AKI prevalence was higher and ARC prevalence lower compared to standard SCr-based eGFR methods. Age- and sex-dependent equations for eGFR (eGFR-Smeets for neonates and eGFR-Pierce for children) best approached measured GFR and should preferably be used to optimize diagnosis of AKI and ARC in this population.

**Graphical Abstract:**

A higher resolution version of the Graphical abstract is available as Supplementary information

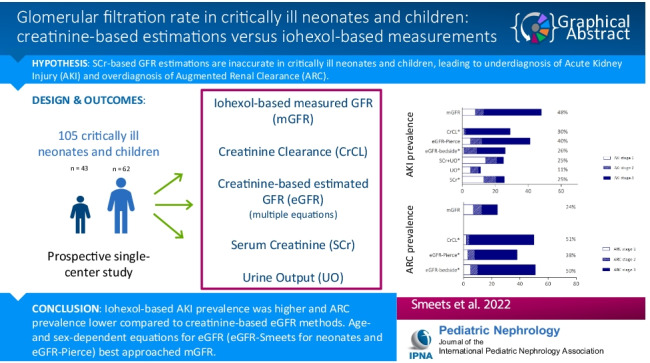

**Supplementary Information:**

The online version contains supplementary material available at 10.1007/s00467-022-05651-w.

## Background

Critically ill children and neonates are at risk for acute kidney injury (AKI), resulting in a sudden derangement of glomerular filtration rate (GFR). This is an independent risk factor for prolonged mechanical ventilation, extended stay in the intensive care unit (ICU), and higher mortality [1]. Augmented renal clearance (ARC), which is enhanced kidney perfusion and glomerular hyperfiltration, is also prevalent in critically ill children [2]. As altered GFR affects fluid and electrolyte management, and requires dose-adaptation drugs cleared by the kidneys, accurate and timely diagnosis of both AKI and ARC is crucial.

The diagnosis of AKI and ARC in clinical care is mostly based on imperfect parameters including serum creatinine (SCr) levels and urine output (UO) [1]. Yet, SCr has several drawbacks and, in addition to glomerular filtration, is also cleared by tubular secretion. Especially in neonates, obtaining accurate GFR estimates is challenging as SCr values reflect maternal creatinine levels and GFR increases in the first days of life [3, 4]. In addition, other endogenous markers for GFR exist, but are rarely used in daily clinical care.

As an alternative to estimated GFR (eGFR) using endogenous biomarkers, GFR can be measured using exogenous substances, such as inulin, radio-isotopes, and iohexol. These markers are inert, non-protein bound, exclusively cleared by glomerular filtration, and free of any tubular handling. Hence, their use to measure GFR (mGFR) results in more accurate determination of GFR than SCr-based estimations and is considered the gold standard for GFR determination [5, 6]. More specifically, iohexol clearances were validated against urinary inulin clearance, and very good agreement was demonstrated in both the adult [7] and pediatric population [8]. To overcome some of the limitations of SCr, and to prevent the need to administer an exogenous compound, creatinine clearance (CrCL), based on Scr urinary creatinine levels and UO, may also be used as it may approach mGFR [9].

In relatively small cohorts of adult ICU patients (18–34 patients), iohexol-based mGFR was compared to SCr-based eGFR [10, 11] or CrCL [12]. In these cohorts, SCr-based eGFR equations showed high inaccuracy when compared to iohexol-based mGFR in critically ill adults [13], resulting in misdiagnosis of AKI and ARC. Because developmental changes in kidney function occur including maturation of GFR and tubular excretion [4], adult findings cannot easily be extrapolated to children. Until now, no mGFR studies were conducted in critically ill children. We hypothesize that SCr-based GFR estimations are inaccurate, leading to underdiagnosis of AKI and overdiagnosis of ARC. Therefore, we aimed to measure GFR using iohexol in critically ill children and term-born neonates. Our primary objective was to compare the prevalence of AKI and ARC between iohexol-based mGFR and SCr, UO, eGFR, and CrCL-based diagnosis. As a secondary objective, we investigated the performance of commonly used SCr-based eGFR methods compared to iohexol-based mGFR as gold standard.

## Methods

### Study design

The methods of our single-center prospective study are described according to the Strengthening the Reporting of Observational Studies in Epidemiology (STROBE) guidelines [14]. Details of the Iohexol for Measuring Renal Function (HERO) study were registered on ClinicalTrials.gov (registration number NCT03946345) before start of the study. The HERO study protocol was approved by the Medical Ethics Review Board (CMO Arnhem-Nijmegen, NL68547.091.18, 2018–5025).

### Setting

The study was performed at the Radboud University Medical Center, Nijmegen, Netherlands, a tertiary teaching hospital providing intensive care to children and neonates. Patients were recruited from the PICU (approximately 570 admissions yearly), and the NICU (200 term newborn admissions yearly), and data were collected between May 2019 and July 2021.

### Patients

Patients were eligible for inclusion if they were below 18 years of age, term-born (≥ 37 weeks of gestation, if < 1 year of age), had a bodyweight of more than 2500 g, and at least one failing organ as defined by a Pediatric Logistic Organ Dysfunction II (PELOD-II) score of 1 or higher (range 0–33) [15]. They also needed to have an indwelling central venous or arterial line already in place for clinical purposes. Exclusion criteria were a known medical history of allergic reaction to injection of iodinated contrast material, receiving kidney replacement therapy or extra corporeal membrane oxygenation (ECMO), and language or cognitive inability of parents/caregivers to understand written and/or oral information. Informed consent needed to be provided by parents or other legal representatives if the child was below 16 years of age. Consent of the child was needed if aged above 12 years of age and medical and cognitive state permitted.

### Diagnostic test

After inclusion, iohexol (Omnipaque® 300 mg/mL, GE Healthcare, Chicago, Illinois, USA) was administered as a single bolus dose adapted to bodyweight as follows: < 10 kg, 1 mL; 10–20 kg, 2 mL; 20–30 kg, 3 mL; 30–40 kg, 4 mL; and ≥ 40 kg, 5 mL [16]. To determine mGFR, blood samples were drawn for analysis of iohexol concentrations at 2, 5, and 7 h after administration. Two-point blood sampling at 2 and 5 h after administration is a validated method for mGFR determination in children [16]. To enhance accuracy as low GFR values were expected, we added another sampling point at 7 h after administration for neonates with a bodyweight of at least 3.5 kg and children older than 28 days of age. SCr levels were determined 2 h after iohexol infusion for eGFR determination to reflect the clinical situation in which SCr is measured at point of care and to correspond to the first blood withdrawal point needed for mGFR, preventing an extra blood withdrawal. Urine was collected for 2 h between 4 and 6 h after infusion of iohexol for urine creatinine levels, to calculate CrCL and corresponding to the second blood withdrawal needed for mGFR at 5 h. We opted to collect urine around the second blood withdrawal, to ensure this corresponds to the elimination phase of iohexol.

### Analytical procedures

SCr was assessed by an enzymatic assay (Creatinine Plus, Roche Diagnostics, Meylan, France). Iohexol plasma concentrations were determined at the Leiden University Medical Centre, Leiden, Netherlands, using a validated high-performance liquid chromatography diode array detection assay [17]. The assay was validated according to the European Medicines Agency bioanalytical method validation guidelines [18].

### Variables

Data were retrieved from the electronic health record and involved demographic data, laboratory results, and observational data of physiological parameters. Demographic data included postnatal age, gestational age, gender, height, and weight. Co-existing conditions and comedication, i.e., vaso-active medication and/or nephrotoxic medication (Supplementary Table S1) and disease severity scores (Pediatric Risk of Mortality III (PRISM-III) [19], Pediatric Index of Mortality 2 (PIM2) [20], and Score for Neonatal Acute Physiology, second version (SNAP-II) [21]) (for neonates only), were collected at the time of inclusion. In addition, the duration of ICU admission was recorded. The amount of urine excreted per hour, corrected for bodyweight (UO), was registered at 8-h intervals.

### Calculation of GFR

Iohexol-based mGFR and eGFR were determined in each patient at a standardized timepoint early at admission, regardless of clinical status or AKI diagnosis, in order to reflect GFR for the entire critically ill population.

### Iohexol-based mGFR

Calculations to determine mGFR in children using iohexol were previously published [16] and are listed in the “Supplementary information” (Equations S1-S4). mGFR based on iohexol plasma clearance was calculated based on the ratio between the administered iohexol dose and the area under the plasma concentration time curve. A slope-intercept method, using the Jødal and Brøchner–Mortensen (JBM) formula with early normalization to 1.73 m^2^ body surface area (BSA), was employed as this method was previously validated in children with CKD [16, 22]. The Haycock formula was used to calculate BSA [23, 24].

### eGFR

eGFR based on serum creatinine was estimated using one formula including different fixed and age-specific coefficients (k):

$$eGFR \left(ml/min/1.73\;m^{2}\right)= k\;\times\;height\;(m)\;/\;SCr\;(mg/dL)$$

First, the frequently used Schwartz equation was used with fixed coefficients reported for children below (*k* = 44) and above 1 year of age (*k* = 41.3) (eGFR-bedside) [25, 26]. For neonates, a coefficient of 31.0 was also tested (eGFR-Smeets) as high accuracy was found using an individual participant data meta-analysis reporting mGFR reference values for healthy term-born neonates (data accepted for publication in Journal of American Society of Nephrology). Additionally, different age- and sex-specific *k* values as reported by Pierce et al., ranging from 33.1 for females of 1 year of age to 48.6 for males of 17 years of age, were used (eGFR-Pierce) [27]. For this equation, the *k* value as reported for 1-year-olds was used for patients below 1 year of age as no specific coefficients for younger children were reported.

CrCL was calculated using 2-h urine collection intervals [28]:

$$CrCl\;(ml/\mathrm{min}/1.73\; m^{2})=\frac{urine\;volume\;\left(ml\right)\;\times urine\;concentration\;creatinine\;(mmol/L)\times1.73}{serum\;creatinine\;\left(\mu mol/L\right)\;\times\;\mathrm{1,000}\;\times\;time \left(min\right)\;\times\;BSA\;(m^{2})}$$

### Definition of AKI and ARC

The prevalence and severity of AKI were determined using mGFR and seven other diagnostic criteria. First, AKI was diagnosed using SCr, UO, and a combination of SCr and UO with the Kidney Disease Improving Global Outcomes (KDIGO) criteria. The KDIGO categories risk (stage 1), injury (stage 2), and failure (stage 3) were defined using median age-specific reference values of enzymatic SCr for neonates [29] and children from 28 days of age [30] to circumvent the lack of baseline SCr values due to unplanned admissions. AKI categories were defined as > 150%, > 200%, and > 300% of median age-specific reference values for SCr, as this approach was previously described by Zwiers et al. to diagnose AKI in critically ill infants [31] Next, in order to enable comparison between mGFR- and eGFR-based diagnosis, eGFR- and mGFR-based prevalence and severity of AKI and ARC was defined based on mGFR and eGFR methods. Three categories of severity were defined based on the mean age-specific reference values for GFR [31]. Again, separate reference values for neonates [32] and children from 1 month of age onward were applied [33]. AKI categories were defined as follows: stage 1, mean_age_ – 1 SD_age_ > GFR ≥ mean_age_ – 1.5 SD_age_; stage 2, mean_age_ – 1.5 SD_age_ > GFR ≥ mean_age_ – 2 SD_age_; and stage 3, GFR < mean_age_ – 2 SD_age_. Because no universal ARC diagnostic criteria exist, they were defined using the opposite AKI criteria by applying + 1, + 1.5, and + 2 SD as cut-off values for the different stages. The prevalence of AKI was reported including all stages as well as dichotomized at stage 2 (only including stage 2 and 3).

### Statistical analysis

#### Sample size

Our sample size was based on the primary study aim. We implemented an expected true proportion of AKI (*p*) of 50% in our calculations, as the SCr-based prevalence in critically ill neonates was 35% [31] and we expected mGFR-based prevalence to be higher. Also, we applied a desired precision (*d*) of 0.1. By using a standard 95% confidence interval (CI), we applied a Z-score of 1.96 resulting in a sample size (*n*) of 98, calculated by n = (Z×p)/d. Accounting for drop-outs, 105 patients were included.

#### Demographics

All demographic data were analyzed for the entire study cohort as well as separately for neonates (≤ 28 days of postnatal age) and children (> 28 days of postnatal age). For continuous variables, data were expressed as median values with interquartile ranges (IQR) or ranges, whereas for categorical variables, numbers and percentages were used.

#### Primary objective: AKI and ARC prevalence and severity

The number of missing values per mGFR and eGFR method was displayed, and prevalence and performance were calculated using available data only. In case only two iohexol concentrations were available, mGFR was calculated using two points. Missing values were not replaced. AKI and ARC prevalences were calculated, and differences in prevalence and severity were compared using a McNemar and McNemar-Bowker Test of Symmetry.

#### Secondary objective: performance of eGFR equations

The comparison of GFR methods for each patient was based on one standardized point in time early at admission, regardless of clinical status or AKI diagnosis. To assess the agreement between several eGFR equations with iohexol-based mGFR, bias and accuracy were calculated. GFR data were analyzed by calculating the difference between eGFR and mGFR per patient and determining the median of this difference (bias). Comparison of eGFR and mGFR values on a group level was performed using the Wilcoxon signed-rank test for paired data. Accuracy was calculated as the percentage of patients having a similar eGFR when compared to mGFR (≤ 30% difference) [13].

Data were analyzed by using SPSS version 25.0.0.1 for Windows (SPSS, Chicago, IL, USA).

## Results

### Patients

**Fig. 1:**
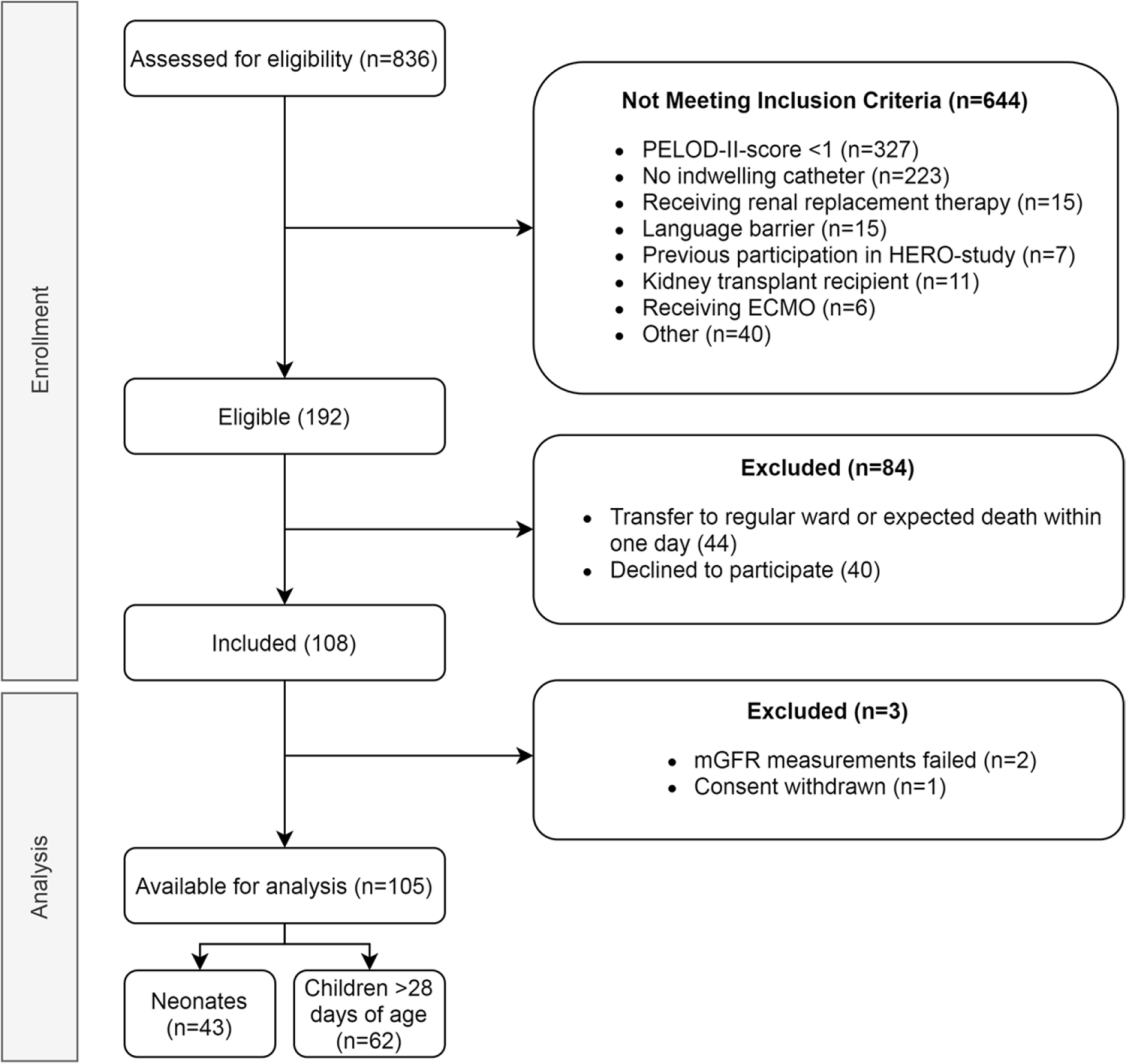
Patient inclusions. Neonates are ≤ 28 days of age, whereas children are > 28 days of age. Abbreviations: PELOD-II-score, Pediatric Logistic Organ Dysfunction version 2 score; HERO, Iohexol for Measuring Renal Function; ECMO, extra-corporeal membrane oxygenation; mGFR, measured glomerular filtration rate

From May 2019 until June 2021, 611 PICU patients and 225 term-born NICU patients were admitted and screened for eligibility (Fig. 1). Of these 836 patients, 192 were eligible. Forty-four patients were eligible but not approached for informed consent due to expected death within 1 day, transfer to regular ward on the same day or based on advice of the treating physician. In total, 108 out of 148 approached patients/parents/legal representatives provided written informed consent. Of these patients, two patients lost their indwelling catheter and for one patient, consent was withdrawn. As no iohexol concentration–time profile could be determined in these patients, they were excluded from further analysis. Of all patients, one (AKI stage 3, according to all different methods) required kidney replacement therapy after study completion, and ten passed away. The patient characteristics are presented for neonates (*n* = 43) and children (*n* = 62) (Table 1). At inclusion, median age was 6.1 years (range 0–17 years) for children and 0 days (range 0–27 days) for neonates. Iohexol was administered after a median duration of 26 h (IQR 16–52) after admission. For 101 patients, mGFR was available. One hundred four patients had SCr values and from 96 patients urinary output data were available. Urinary CrCL values were available for 81 patients.

**Table 1:** Patient characteristics

	TOTAL	Neonates (< 28 days of age)	Children (> 28 days of age)
Number of patients	105	43	62
Male sex^#^	64 (61%)	24 (56%)	40 (65%)
Age:			
- Years^†^ - Days^†^	0.0 (0.0–17.2) 102 (0–6274)	0.0 (0.0–0.1) 2 (0–27)	6.1 (0.1–17.2) 2232 (31–6274)
Weight (kg)*	6.5 (3.5–29.8)	3.5 (2.9–3.8)	21.5 (8.2–40.0)
Height (cm)*	63 (51–133)	51 (49–52)	114 (73–152)
Primary diagnosis^#^			
- Shock - Cardiovascular problems - Respiratory problems - Surgical problems or trauma - Central nervous system problems	4 (3.8) 21 (20.0) 49 (46.7) 16 (15.2) 14 (13.3)	0 (0.0) 7 (16.3) 26 (60.5) 8 (18.6) 2 (4.7)	4 (6.5) 14 (22.6) 23 (37.1) 8 (12.9) 12 (19.4)
-Sedation or pain management	1 (1.0)	0 (0.0)	1 (1.6)
Total PELOD-II score^†^	5 (1–18)	5 (2–16)	5 (1–18)
PRISM-III score^†^	-	-	6 (0–26)
PIM2 probability score^†^	-	-	0.039 (0.016–0.98)
SNAP-II-score^†^	-	0 (0–68)	-
Patients using vaso-active drugs^#^	54 (51%)	14 (32%)	40 (65%)
Patients using nephrotoxic drugs^#^	51 (49%)	16 (37%)	35 (56%)
Time between admission and start of study (h)*	26 (16–52)	28 (16–45)	26 (16–56)
Duration of total stay at ICU (h)*	190 (101–300)	181 (98–300)	196 (104–304)

*PICU*, pediatric intensive care unit; *NICU*, neonatal intensive care unit; IQR, interquartile range; *PELOD-II-score*, Pediatric Logistic Organ Dysfunction version 2 score; *PRISM-III-score*, Pediatric Risk of Mortality score, third version; *PIM2-score*, Pediatric Index of Mortality score, second version; *SNAP-II-score*, Score for Neonatal Acute Physiology, second version; *, median (IQR); ^†^, median (range); ^#^, no. (%)

### AKI prevalence

**Fig. 2:**
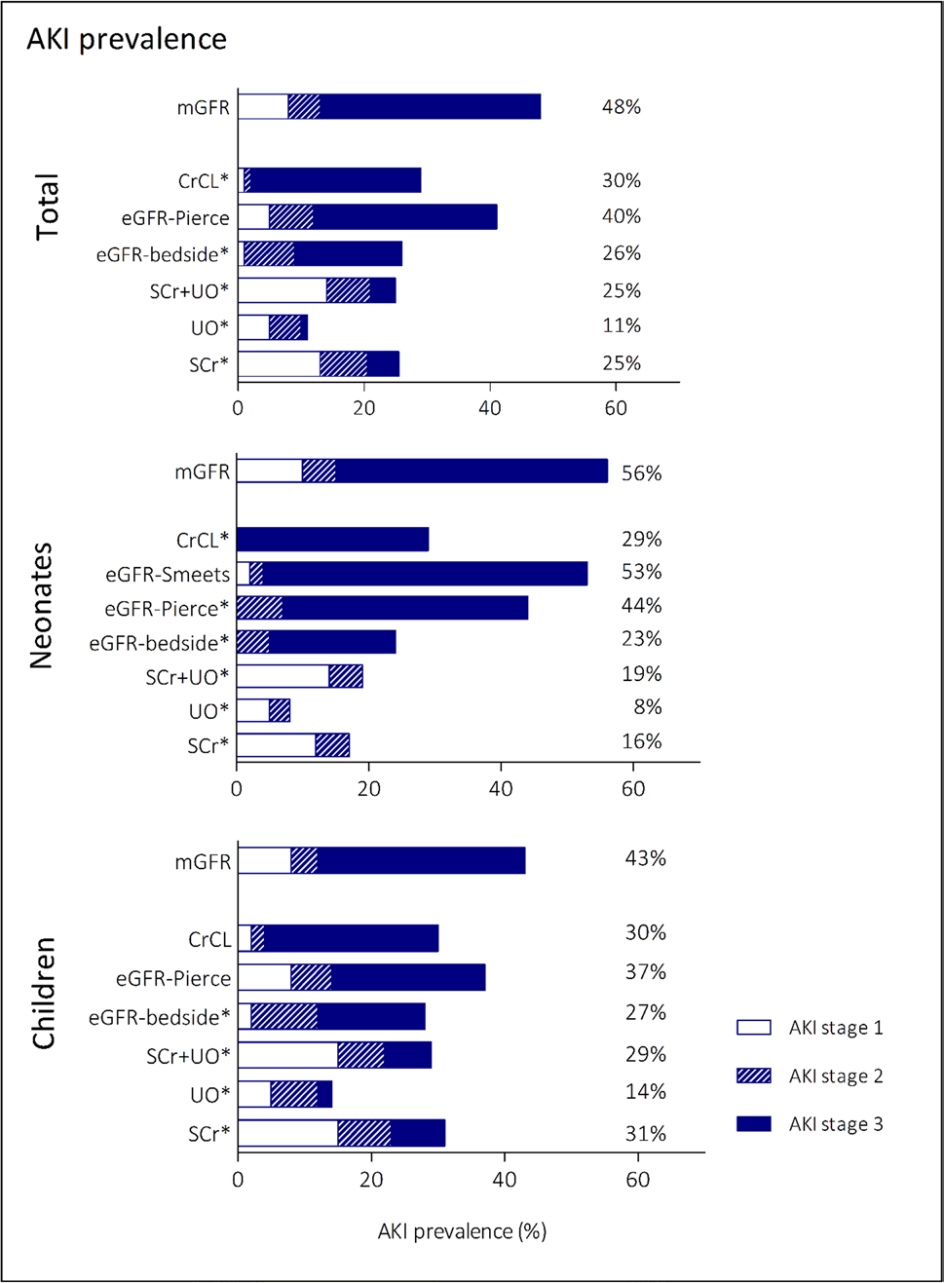
Prevalence of AKI using mGFR and different eGFR based methods*.* Statistical significance differences (*p* < 0.05) based on McNemar Bowker test for symmetry between eGFR and mGFR defined AKI are indicated by *. Abbreviations: SCr, serum creatinine; UO, urine output; mGFR, measured glomerular filtration rate; eGFR, estimated glomerular filtration rate; CrCL, creatinine clearance; AKI, acute kidney injury

Iohexol-based (mGFR) AKI prevalence was 48% compared to 11–40% for KDIGO- or eGFR-based diagnosis (Fig. 2). For neonates, this was 56% for mGFR and 8–53% for KDIGO- or eGFR-based diagnosis. Also, in children, a similar pattern was observed with 43% mGFR-based prevalence compared to 14–37% for other methods. Staging of AKI differed significantly between the methods (McNemar-Bowker test, *p* =  < 0.001–0.037), except between mGFR and eGFR-Pierce for both neonates and children (*p* = 0.127), and, in neonates, between mGFR and eGFR-Smeets (*p* = 0.236). When dichotomized at stage 2, the observed trend was similar. mGFR-based AKI prevalence was 40% compared to 6–35% for KDIGO- or eGFR-based diagnosis. For neonates, this was 46% for mGFR-based diagnosis versus 3–51% for KDIGO- or eGFR-based diagnosis. In children, AKI prevalence was 35% when diagnosis was based on mGFR, compared to 9–29% for other methods.

### ARC prevalence

**Fig. 3:**
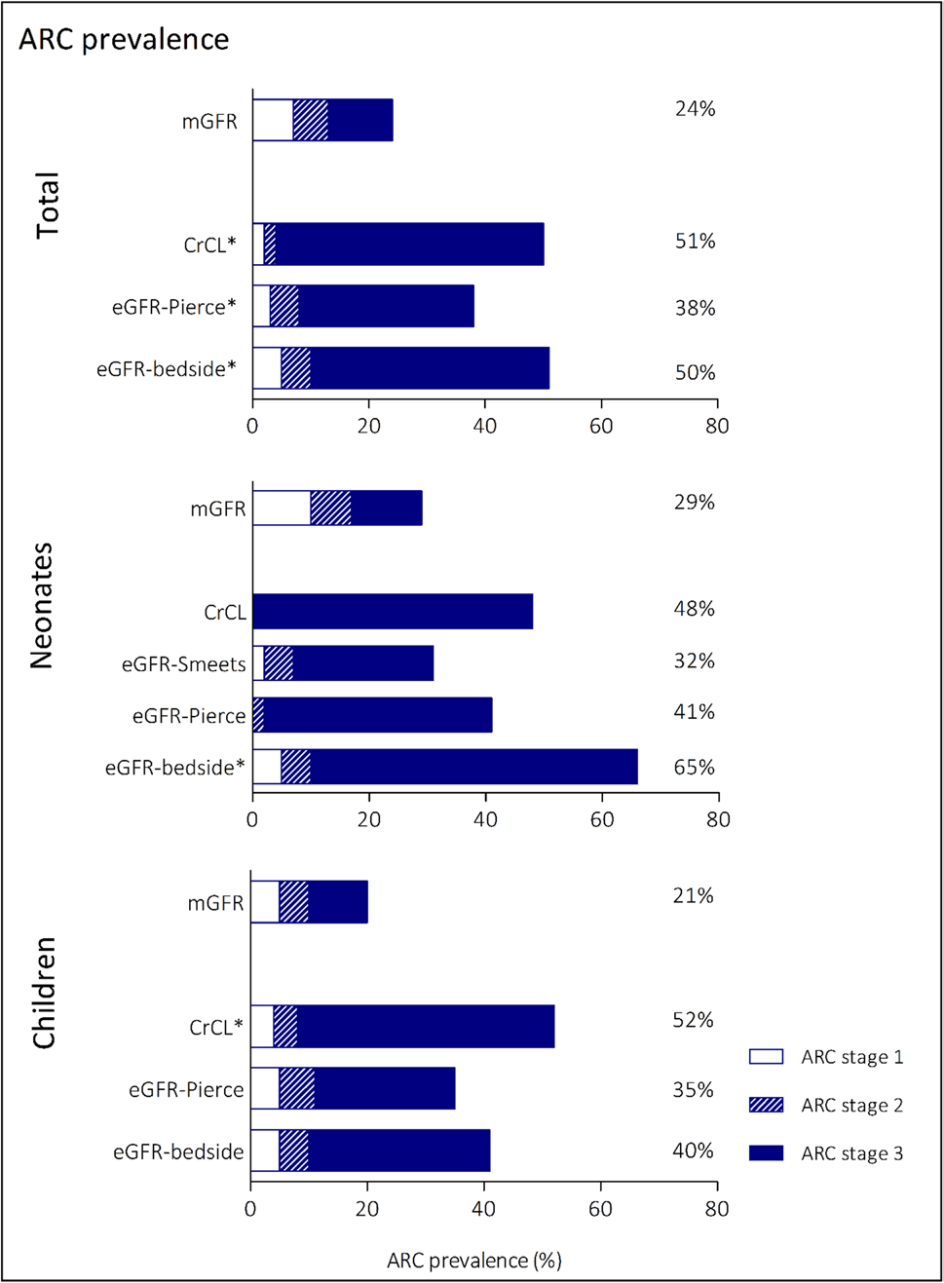
Prevalence of ARC using mGFR and different eGFR-based methods. Statistically significant differences (*p* < 0.05) based on McNemar Bowker test for symmetry between eGFR and mGFR defined ARC are indicated by *. Abbreviations: mGFR, measured glomerular filtration rate; eGFR, estimated glomerular filtration rate; CrCL, creatinine clearance; ARC, augmented renal clearance

ARC prevalence was 24% when based on mGFR, as opposed to 38–51% using eGFR methods (Fig. 3). In neonates, this was 29% for mGFR and 32–65% for eGFR, whereas in children, this was 21% for mGFR and 35–52% for eGFR. Again, staging of ARC differed significantly between mGFR and eGFR (McNemar Bowker test, *p* =  < 0.001–0.018).

### Performance of eGFR equations

Median mGFR was 40.6 (IQR 26.4–92.9) ml/min/1.73 m^2^. In children, median mGFR was 80.7 (IQR 42.3–114.7) versus 29.2 (IQR 22.3–35.0) ml/min/1.73 m^2^ in neonates (Table 2). For all methods, with the exception of eGFR-Pierce in neonates and eGFR-Smeets in neonates, mGFR was significantly lower than eGFR (*p* =  < 0.001–0.016). Median difference between eGFR and mGFR ranged from 0.0 (IQR − 6.4 to 5.2) ml/min/1.73 m^2^ for eGFR-Smeets in neonates up to 28.7 (IQR 5.6–88.1) ml/min/1.73 m^2^ for CrCL in children. Accuracy varied accordingly between 19.4 and 74.4%. Analysis was repeated for patients without AKI only, which yielded similar results (Supplementary Table S2).

**Table 2:** Agreement between methods

	Median GFR (IQR) in ml/min/1.73 m^2^	Median difference between eGFR and mGFR (IQR) in ml/min/1.73 m^2^	*P* value^A^	Accuracy (%)
TOTAL				
mGFR	40.6 (26.4–92.9)			
eGFR-bedside	69.2 (37.0–115.2)	11.0 (0.69–36.7)	< 0.001	41.9
eGFR-Pierce	59.7 (29.5–105.2)	3.9 (− 6.0 to 20.5)	0.002	59.4
CrCL	71.1 (28.9–146.9)	20.1 (− 4.7 to 57.0)	< 0.001	22.9
Children				
mGFR	80.7 (42.3–114.7)			
eGFR-bedside	100.7(67.4–131.1)	17.0 (− 4.6 to 49.3)	< 0.001	40.3
eGFR-Pierce	89.8 (60.9–127.9)	7.1 (− 10.5 to 30.0)	0.016	50.0
CrCL	116.4 (48.9–210.8)	28.7 (5.6 to 88.1)	< 0.001	19.4
Neonates				
mGFR^#^	29.2 (22.3–35.0)			
eGFR-bedside^#^	37.3 (26.9–51.9)	9.9 (1.9–20.0)	< 0.001	44.2
eGFR-Pierce	29.3 (21.0–41.3)	2.0 (− 3.4 to 9.2)	0.055	67.4
eGFR–Smeets^#^	26.3 (18.9–36.5)	0.0 (− 6.4 to 5.2)	0.847	74.4
CrCL	33.4 (23.4–58.7)	7.3 (− 4.9 to 20.2)	0.014	27.9

Median GFR and median bias with corresponding IQR are displayed in mL/min/1.73 m^2^. ^A^ Comparison of mGFR and eGFR using the Wilcoxon signed-rank test. Abbreviations: *eGFR*, estimated glomerular filtration rate; *mGFR*, measured glomerular filtration rate; *CrCL*, creatinine clearance; *IQR*, interquartile range. ^#^Performance in critically ill neonates as previously reported by Smeets et al. (unpublished data)

## Discussion

### Key results

We demonstrated that the prevalence of AKI and ARC between iohexol-based mGFR and eGFR differed, as hypothesized. AKI prevalence was higher (49%) when using mGFR compared to KDIGO- or eGFR-based diagnosis (11–40%). Similarly, mGFR-based ARC prevalence was lower (24%) compared to diagnosis based on various eGFR methods (38–51%). Also, all clinically used eGFR methods presented significant biases and poor accuracy when compared to mGFR values, thereby significantly overestimating GFR (between 2.0 and 28.7 ml/min/1.73 m^2^). Of all equations, in children, the eGFR-Pierce and in neonates, the eGFR-Smeets demonstrated the highest accuracy and lowest bias and could therefore be used in clinical care to improve GFR estimations in critically ill children and neonates.

### Interpretation of AKI and ARC prevalence

Our data are innovative; as to the best of our knowledge, this is the first study in critically ill children and/or term-born neonates, and our cohort is by far the largest of critically ill patients, as adult studies included up to 66 patients. Additionally, both AKI and ARC were assessed using mGFR in the same ICU population. Our cohort is representative of other pediatric ICU cohorts, as our SCr and/or UO-based AKI prevalence (23–29%) is similar to previously published AKI prevalences in critically ill children (27%) and in infants (< 1 year of age) (35%) [1, 31]. The lack of pediatric mGFR studies prevents comparison to other pediatric mGFR data. In the large AWAKEN cohort, in nearly one in five pediatric AKI patients, SCr was not increased, but diagnosis was based on reduced UO [34]. Also, in our cohort, in 24% of mGFR-based AKI patients SCr levels were not increased. Yet, by including UO as diagnostic criterion for AKI, only two additional patients were diagnosed compared to SCr-based criteria in our cohort (data not shown).

ARC prevalence in critically ill children has only been reported using vancomycin clearance as a surrogate of mGFR [35]. By defining ARC as vancomycin clearance of ≥ 130 ml/min/1.73 m^2^ regardless of age, 12% of 250 critically ill children were diagnosed with ARC, compared to 24% in our cohort. A limitation of this study was the use of an age-independent cut-off for clearance, ignoring age-related GFR changes. This could have led to an underestimation of ARC prevalence. When AKI and ARC patients are not classified as such, drug dosing might be suboptimal leading to therapy failure or toxicity. This poses patients at risk for adverse outcomes [2, 36]. In our cohort, neither ventilator-free days nor duration of stay differed between mGFR-based AKI patients who would have been missed using KDIGO-based diagnosis only, compared to both KDIGO- and mGFR-based AKI diagnosis (data not shown). This could be due to the relatively short follow-up and limited sample size of our cohort. Whether toxicity or therapy failure could be diminished by using mGFR-based diagnosis remains to be studied in a larger cohort.

### Interpretation of performance of eGFR methods

Our results illustrate the difference in performance of different eGFR methods and highlight the importance of using age-specific SCr-based eGFR equations (i.e., eGFR-Smeets for neonates and eGFR-Pierce for children) as these give the best approximation of GFR. To date, no mGFR validation studies in critically ill children have been performed, preventing comparison of our results to other pediatric cohorts. However, seven studies in critically ill adults (*n* = 18–66) investigated the performance of several eGFR equations. Similar to our results, multiple SCr-based eGFR equations displayed high biases and low accuracy (23–60%, dependent on equation used) when compared with mGFR values [11, 13, 28, 37–40].

The inaccuracy of SCr-based eGFR equations can be understood by understanding the pharmacokinetic properties of this marker, next to the other drawbacks of SCr as mentioned in the introduction. Compared to iohexol, the volume of distribution of creatinine is larger as it distributes into the total body water (TBW), whereas iohexol is only distributed in the extracellular volume (ECV) [41]. Correspondingly, because the ECV comprises around one-third of the TBW, creatinine half-life is around three times higher than iohexol half-life [42]. eGFR based on creatinine is therefore reflecting GFR over a longer (preceding) period. Consequently, iohexol-based mGFR is able to detect changes in GFR earlier than SCr-based eGFR. Even though the performance of all SCr-based eGFR equations is limited in critically ill patients, there are significant differences in performance between the different eGFR equations. Attention should therefore be paid to use and implement the best equation for each patient population with mGFR. Using the appropriate formula (i.e., eGFR-Smeets for neonates and eGFR-Pierce for children) could improve GFR estimations in clinical care.

Because of the limitations of SCr, cystatin C (cysC) has been proposed as an alternative marker of GFR. CysC has consistent stable plasma levels from 1 year of age, and its levels are independent of muscle mass [43]. Also, cysC is not secreted by the tubules, and distributed in the ECV only, explaining the shorter half-life compared to creatinine. Therefore, the value of cysC as a marker for GFR in the neonatal and pediatric population has been investigated by many and is considered promising. However, cysC levels are significantly affected by thyroid disorders [44] and corticosteroids [45]. Whether cysC-based eGFR equations are of value in critically ill neonates and children remains to be validated.

### Limitations

Our study has some limitations. First, we considered three-point iohexol clearance as the gold standard for mGFR as opposed to a rich sampling schedule, as optimal sampling schemes with more than three sampling points only marginally increased accuracy and precision [17, 46, 47]. We therefore believe this did not affect our conclusions. Additionally, by using an extra sampling point at 7 h, we optimized accuracy to account for low GFR. Because critical illness is highly dynamic and GFR shows an evolution over time, using single-bolus iohexol plasma clearance will only allow for the calculation of *mean* GFR over the sampling period, in our case 7 h. Consequently, GFR results will become available after this period of time. When using continuous (low-dose) iohexol infusion, this problem might be circumvented as steady state is immediately obtained after a loading dose is administered [48], and clearance can be calculated at point of care. However, administering the very small neonatal doses in a continuous manner is impractical with the current infusion pumps, and occupying a lumen of an IV line for the entire day is undesirable. Hence, we used single-bolus infusion of iohexol in our cohort. Next to the “real” differences between SCr-based eGFR and mGFR discussed above, these limitations could also have contributed to the observed differences between eGFR and mGFR.

### Future perspectives

Routine measurements of iohexol-based GFR are increasingly used in standard of care, especially in (pediatric) CKD patients, in the kidney transplant setting, and in oncology [16, 17, 49]. The use of iohexol as a marker for GFR is safe [7, 50] and iohexol has a low interaction potential [49]. This enables its use in the intensive care setting where patients receive multiple therapies. Also, the calculation needed to calculate GFR based on measured concentrations is easy to implement in clinical support systems as demonstrated by Zwart et al. [17], making mGFR results directly visible for treating physicians. Understandably, all iohexol adult studies advocated the use of routine iohexol measurements in the intensive care setting [11, 13, 28, 37–40].

Contrary to these adult studies, we believe, at this point in time, that the added value of increased accuracy of GFR determination using iohexol plasma clearance in the neonatal and pediatric intensive care setting will not outweigh the disadvantages of the cumbersome procedure. In addition, the lack of extensive availability to analyze iohexol plasma concentrations at the point of care and to perform pharmacokinetic analysis of data 24/7 in most centers will hamper its direct implementation. However, for certain patients in which the use of SCr as a marker for GFR is severely hampered (e.g., low muscle mass), it might be worthwhile to determine mGFR to link SCr levels to GFR. Also, future perspectives regarding mGFR determination are promising. To circumvent the impracticality of GFR measurements, the plasma clearance of iohexol can be calculated using modelling and simulation approaches based on few blood withdrawals within 4 h after administration. This method of GFR determination has already been implemented in adult kidney transplantation care [17] and would be of great value in the intensive care setting. Furthermore, novel technologies provide opportunities for the continuous monitoring of GFR by transdermal measurement of a fluorescent GFR marker [51, 52]. Without losing accuracy and precision, these methods could facilitate the use of rapid and reliable determination of GFR at the bedside without the need for indwelling catheters.

Until iohexol-based GFR measurements using sparse sampling schedules are available for critically ill children and neonates at point of care, including the best eGFR equation in electronic decision support systems that present eGFR when creatinine values and height are available, is of great importance. According to our findings, we propose the eGFR-Pierce for children and eGFR-Smeets for neonates. As GFR-adjusted dosing has been shown to optimize drug target concentrations for drugs cleared by the kidneys [53], using these eGFR equations could prevent over- or under-dosing of drugs cleared by the kidney and might optimize fluid and electrolyte management in AKI or ARC patients.

## Supplementary Information

Below is the link to the electronic supplementary material.

Supplementary file1 (DOCX 18 kb)

Graphical Abstract (PPTX 169 kb)

## Data Availability

The datasets used and/or analyzed during the current study are available from the corresponding author on reasonable request.

## Abbreviations

AKI: Acute kidney injury
ARC: Augmented renal clearance
BSA: Body surface area
BUN: Blood urea nitrogen
CI: Confidence interval
CKD: Chronic kidney disease
CL: Clearance
CrCL: Creatinine clearance
cysC: Cystatin C
ECMO: Extracorporeal membrane oxygenation
eGFR: Estimated glomerular filtration rate
GFR: Glomerular filtration rate
ICU: Intensive care unit
JBM: Jødal and Brøchner–Mortensen
k: Coefficient
KDIGO: Kidney Disease Improving Global Outcomes
mGFR: Measured glomerular filtration rate
NICU: Neonatal intensive care unit
PELOD-II: Pediatric Logistic Organ Dysfunction, second version
PICU: Pediatric intensive care unit
PIM2: Pediatric Index of Mortality, second version
PRISM-III: Pediatric Risk of Mortality, third version
SCr: Serum creatinine
SNAP-II: Score for Neonatal Acute Physiology, second version
STROBE: Strengthening the Reporting of Observational Studies in Epidemiology
UO: Urine output
Vd: Volume of distribution
