# Supplementary material for: Glomerular filtration rate in critically ill neonates and children: creatinine-based estimations versus iohexol-based measurements
Source: Pediatr Nephrol. 2022 Aug 2;38(4):1087–97. doi: 10.1007/s00467-022-05651-w (PMC9925555; doi:10.1007/s00467-022-05651-w)
Supplement: Supplementary file 1 — (DOCX 18 kb) [file 467_2022_5651_MOESM1_ESM.docx]

# Supplementary material

This appendix has been provided by the authors to give readers additional information about their work.

Supplementary content to: *Diagnosis of acute kidney injury and augmented renal clearance in critically ill children and neonates using iohexol plasma clearance.*

Supplementary tables and equations

Table S1

| **Vaso-active drugs** | **Nephrotoxic drugs** |
| --- | --- |
| Dobutamin  Dopamin  Ephedrine  Enoximone  Epinephrine  Isoprenaline  Levosimendan  Midodrin  Milrinone  Nitroglycerin  Nitroprusside  Norepinephrine  Phenylephrine  Vasopressin | Acyclovir  Amikacin  Amphotericin  Captopril  Carboplatin  Cidofovir  Cisplatinum  Cyclophosphamide  Cyclosporin  Enalapril  Enalaprilat  Ethacrynic acid  Flucytosine  Foscarnet  Furosemide  Ganciclovir  Gentamicin  Hydrochlorothiazide  Ibuprofen  Ifosfamide  Indomethacin  Ketorolac  Methotrexate  Penicillin  Ramipril  Sirolimus  Tacrolimus  Tobramycin  Vancomycin |

**Table S1:** List of vaso-active and nephrotoxic drugs.

Table S2

| **Method of GFR determination** | **Median GFR (IQR)**  **In ml/min/1.73m^2^** | | **Median difference between eGFR and mGFR (IQR)**  **in ml/min/1.73m^2^** | | | | **P-value^A^** | | **Accuracy (%)** | |  |  |
| --- | --- | --- | --- | --- | --- | --- | --- | --- | --- | --- | --- | --- |
| **TOTAL** | | | | | | | | | | | |  |
| mGFR | 87.1 (36.5-117.4) | |  | | | |  | |  | |  |  |
| eGFR - bedside | 103.1 (52.7-131.7) | | 11.0 (-3.0-43.4) | | | | 0.001 | | 47.2 | |  |  |
| eGFR - Pierce | 94.7 (41.7-128.1) | | 4.4(-11.0-24.0) | | | | 0.116 | | 60.4 | |  |  |
| CrCL | 140.7 (52.5-231.0) | | 47.9(8.2-97.0) | | |  | 0.000 | | 18.9 | |  |  |
| **Children** |  |  | |  |  | | |  | |  | | |
| mGFR | 112.1 (92.9-133.4) | |  | | | |  | |  | |  |  |
| eGFR - bedside | 118.1 (109.3-185.1) | | 12.3(-18.5-62.5) | | | | 0.031 | | 48.5 | |  |  |
| eGFR - Pierce | 114.2 (97.1-150.8) | | 5.8 (-18.3-40.9) | | | | 0.228 | | 60.6 | |  |  |
| CrCL | 204.0 (137.6-242.3) | | 69.2 (23.9-142.0) | | |  | 0.000 | | 18.2 | |  |  |
| **Neonates** |  |  | |  |  | | |  | |  | | |
| mGFR | 31.1 (27.2-43.2) | |  | | | |  | |  | |  |  |
| eGFR - bedside | 39.1 (33.6-71.6) | | 10.9 (1.3-30.5) | | | | 0.001 | | 45.0 | |  |  |
| eGFR - Pierce | 31.7 (25.5-56.4) | | 3.3 (-5.8-13.0) | | | | 0.135 | | 60.0 | |  |  |
| eGFR – Smeets^#^ | 27.6 (23.7-50.5) | | 0.4 (-7.5-8.7) | | | | 0.852 | | 70.0 | |  |  |
| CrCL | 43.0(28.5-82.6) | | 12.6 (-6.3-37.7) | | |  | 0.087 | | 20.0 | |  |  |

**Table S2:** Agreement between methods for patients without AKI only (n=53). Median GFR and median bias with corresponding IQR are displayed in mL/min/1.73 m^2^. ^A^ Comparison of mGFR and eGFR using the Wilcoxon signed rank test. Abbreviations: eGFR: estimated glomerular filtration rate, mGFR: measured glomerular filtration rate; CrCL: creatinine clearance; IQR; interquartile range. ^#^Performance in critically ill neonates as previously reported by Smeets et al(unpublished data).

Supplementary equations

$${CL}_{I} = I/ expA/\alpha$$

**Equation S1**: Calculation of clearance, based solely on slow phase of elimination curve (CL_I_). I: dose of iohexol in milligrams, expA: intercept of the slow curve (elimination phase, log concentration vs. time), α: corresponding slope.

$${CL}_{I, BSA} ={CL}_{I} x 1.73/BSA$$

**Equation S2:** Calculation of BSA-corrected clearance, based solely on slow phase of elimination curve. CL_I_: clearance based on slow phase of elimination curve. BSA: body surface area

$$f_{BSA}=0.00185BSA^{-0.3}$$

**Equation S3:** Calculation of a BSA corrected factor. BSA: body surface area.

$${CL}_{corrected}= {CL}_{I}, BSA /1+f_{BSA} x {CL}_{I}, BSA$$

**Equation S4:** Calculation of clearance, corrected for BSA, in ml/min/1.73m^2^. CL_I, BSA_: clearance based on slow phase of elimination curve, corrected for BSA, BSA: body surface area, f_BSA_: BSA corrected factor
