# Supplementary material for: Glomerular filtration rate in critically ill neonates and children: creatinine-based estimations versus iohexol-based measurements
Source: Pediatr Nephrol. 2022 Aug 2;38(4):1087–97. doi: 10.1007/s00467-022-05651-w (PMC9925555; doi:10.1007/s00467-022-05651-w)
Supplement: Supplementary file 2 — (PPTX 169 kb) [file 467_2022_5651_MOESM2_ESM.pptx]

## Slide 1
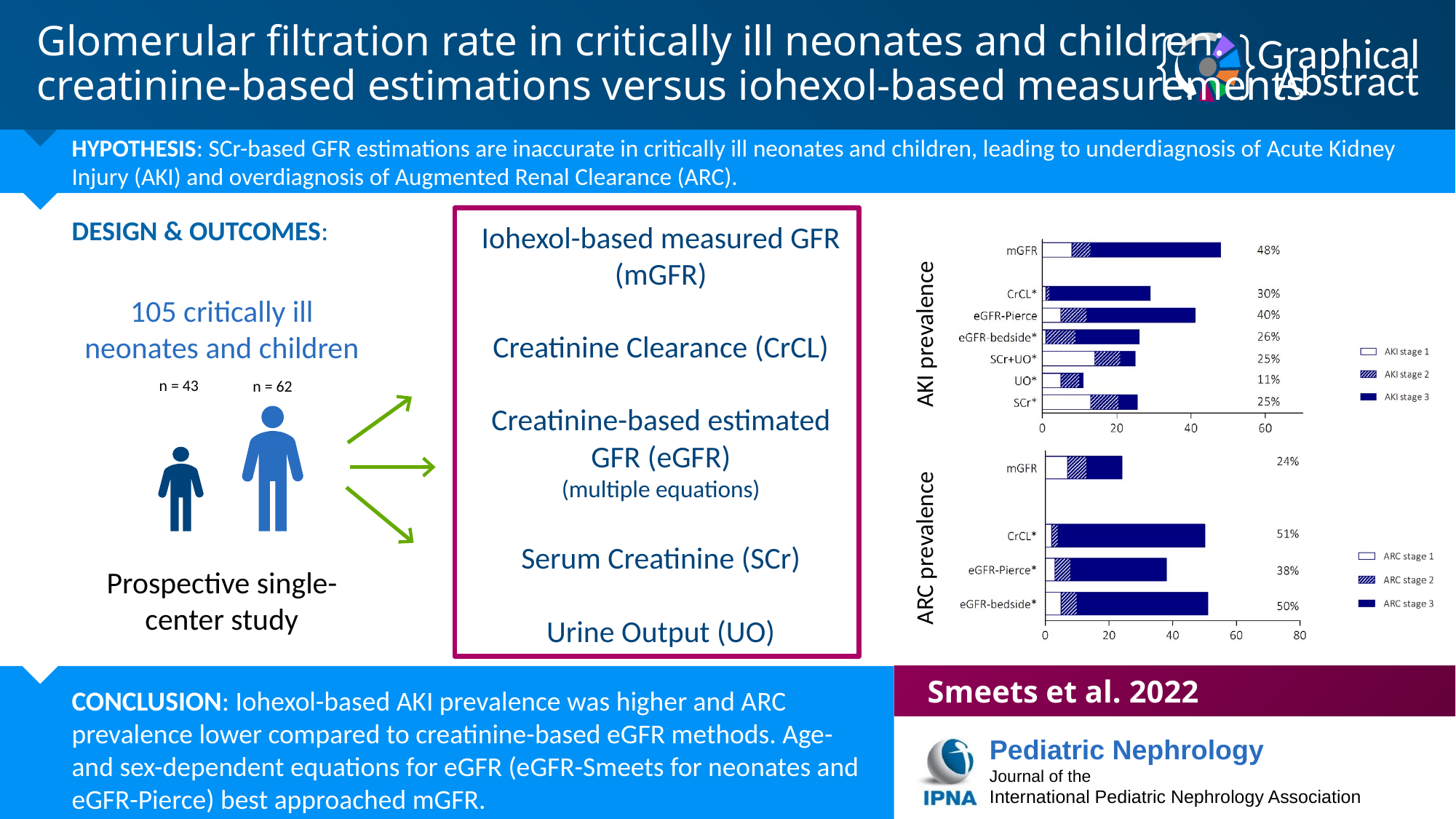

Glomerular filtration rate in critically ill neonates and children:
creatinine-based estimations versus iohexol-based measurements
HYPOTHESIS: SCr-based GFR estimations are inaccurate in critically ill neonates and children, leading to underdiagnosis of Acute Kidney Injury (AKI) and overdiagnosis of Augmented Renal Clearance (ARC).
DESIGN & OUTCOMES:
Iohexol-based measured GFR (mGFR)
Creatinine Clearance (CrCL)
Creatinine-based estimated GFR (eGFR)
(multiple equations)
Serum Creatinine (SCr)
Urine Output (UO)
105 critically ill neonates and children
AKI prevalence
n = 43
n = 62
ARC prevalence
Prospective single- center study
Smeets et al. 2022
CONCLUSION: Iohexol-based AKI prevalence was higher and ARC prevalence lower compared to creatinine-based eGFR methods. Age- and sex-dependent equations for eGFR (eGFR-Smeets for neonates and eGFR-Pierce) best approached mGFR.
